# Extended Cheese Whey Fermentation Produces a Novel Casein-Derived Antibacterial Polypeptide That Also Inhibits Gelatinases MMP-2 and MMP-9

**DOI:** 10.3390/ijms222011130

**Published:** 2021-10-15

**Authors:** Maria Isabel Santos, Ana Lima, Joana Mota, Patrícia Rebelo, Ricardo Boavida Ferreira, Laurentina Pedroso, Maria Adélia Ferreira, Isabel Sousa

**Affiliations:** 1 Linking Landscape, Environment, Agriculture and Food (LEAF), Instituto Superior de Agronomia, University of Lisbon, Tapada da Ajuda, 1349-017 Lisboa, Portugal; agusmaolima@gmail.com (A.L.); joana.mota.p@gmail.com (J.M.); patricia.a.o.rebelo@gmail.com (P.R.); rbferreira@isa.ulisboa.pt (R.B.F.); laurentina.pedroso@ulusofona.pt (L.P.); masssferreira@netcabo.pt (M.A.F.); isabelsousa@isa.ulisboa.pt (I.S.); 2 Faculty of Veterinary Medicine, Universidade Lusófona de Humanidades e Tecnologias, Campo Grande, 376, 1749-024 Lisboa, Portugal

**Keywords:** whey, fermentation, MMP-9, MMP-2, antibacterial activity, MMP inhibitor, peptide

## Abstract

Our previous works produced a whey fermentation methodology that yielded antibacterial activity and potential inhibition of matrix metalloproteases (MMP)-2 and -9. Here, we evaluated if these activities were due to fermentation-produced peptides. Prolonged fermentation was carried out in the presence of our specific lactic acid bacteria (LAB) consortium. LAB fermentation yielded a total of 11 polypeptides, which were predominantly produced after 6 days of fermentation. One which was derived from beat casein presented a particularly high antibacterial activity against food pathogenic bacteria and was more effective than standard food disinfectants. This polypeptide was further studied and was also found to be active against several strains of pathogenic bacteria, including methicillin-resistant *Staphylococcus aureus* (MRSA), in a dose-dependent manner. It also inhibited MMP-2 and MMP-9 whilst reducing HT29 cancer cell migration in vitro. Overall, this novel whey-derived polypeptide presents dual antibacterial and anti-inflammatory activity, revealing a strong potential to be used in functional foods or as a nutraceutical. Its identification and further characterization can open novel perspectives in the field of preventive/curative diets related to gut microbiota, gut inflammation, and cancer prevention, particularly if used in in vivo studies.

## 1. Introduction

Whilst milk proteins have long been recognized as major sources of bioactive and nutritional components, an increasing body of research in bioactive peptides derived from milk and whey has been gradually opening up a novel range of possibilities in functional foods and nutraceuticals [1,2,3,4,5,6]. Some of the biological functions of milk proteins are only revealed upon proteolytic action, which produces peptides that directly influence numerous biological processes evoking behavioral, gastrointestinal, hormonal, immunological, neurological, and nutritional responses [7]. According to Jabbari et al. (2012) [8], these peptides have great potential to be used in health-enhancing nutraceuticals; however, most of them are difficult to produce with cost-effective technologies. One possible way to overcome this would be inducing proteolysis through lactic acid bacteria (LAB) fermentation. In our previous work [9], the potential of a specific mesophilic LAB starter mix from a Portuguese cheese whey factory was evaluated for its viability as a food bio-preservative. We found that prolonged low-cost fermentation (5-day fermentation) technology using a specific starter could induce high antibacterial activity against a broad range of strains, including *Listeria monocytogenes, Salmonella enterica* Goldcoast, and *Escherichia coli* O157. This was found when using a fermented disinfecting agent for minimally processed salads; this fermented whey was just as effective as chlorine [10,11]. Due to the high proteolytic activity of the LAB consortium, it is quite feasible that the antibacterial activity observed could be the result from hydrolysis-derived peptides and not just lactic acid build-up. Being produced by lactic acid bacteria, fermentation peptide products are generally accepted as safe (GRAS with grade 1 status) and may hold the advantage of promoting (as a probiotic) a healthy gut microbiome. Therefore, isolation and identification of these peptides produced by prolonged whey fermentation may have high potential in the area of food bioactive compounds and nutraceuticals.

Furthermore, our preliminary results suggested that our fermented whey can reduce the activity of matrix metalloproteinases (MMPs), particularly gelatinases MMP-2 and MMP-9, which are important key players in inflammatory bowel diseases (IBDs) [12,13,14,15,16,17,18,19,20,21]. A combination of antibacterial and anti-gelatinase activities could be of particular interest as it has been reported that an imbalance in normal gut microbiota is strongly linked to gastrointestinal conditions such as IBD and irritable bowel syndrome (IBS), which in turn opened up an exciting new field of studies, both in medical as well as food research. Although there are, to our knowledge, no reports on the effect of whey peptides on MMP-2 and -9, recent studies demonstrate that MMP-9 expression in the colon causes alterations in the fecal microbiome and has a serious impact on the pathogenesis of bacterial-induced colitis in mice [22]. In fact, it has been reported that gut inflammation (which directly involves MMP-2 and -9) often involves interactions between pathogenic bacteria and inflammatory responses in the gut lining and also the gut microbiome [7,8]. Hence, the evaluation of these peptides’ potential for inhibiting bacterial growth and reducing inflammatory gut-related processes could be of significant importance. Under this context, our main goal in this work was to identify, isolate, and characterize the activity of the peptides produced in our prolonged cheese whey fermentation and to determine their potential as antibacterial and anti-inflammatory agents.

With the increasing health concerns about multi-drug-resistant bacterial diseases, gut inflammatory diseases, gut microbiome, and foodborne pathogens, this type of bioactive compound could be a turning point in the field of health foods and nutrition.

## 2. Results and Discussion

**Figure 1:**
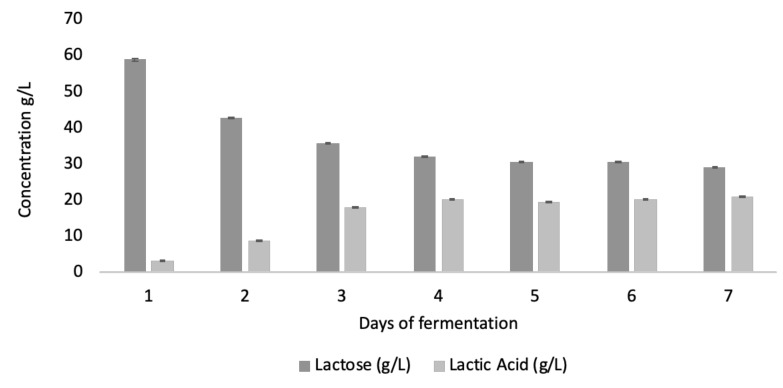
Lactose and lactic acid quantified in whey fermented over 6 days with *Lactococcus lactis* subsp. *lactis*, *Lactococcus lactis* subsp. *cremoris*, *Lactococcus lactis* subsp. *lactis* biovar *diacetylactis*, *Streptococcus thermophilus*, and *Lactobacillus delbrueckii* subsp. *bulgaricus*. Values are an average of at least 3 replicates ± SD.

Whey proteins stand out for their high nutritional value in terms of biological value; nonetheless, these proteins can contain sequences encoded in their primary structure which can be released through proteolysis during digestion. These sequences have been extensively reviewed [23,24,25] and shown to present several types of bioactivities, with antibacterial activity being the most studied, particularly after digestive hydrolysis. Although less studied, the production of bioactive peptides during lactic acid fermentation can also arise from either the proteolysis itself or from the bacteria species used. Most milk proteins are known to produce bioactive peptides upon whey and milk fermentation, such as lactoferrin (lactoferricin), lactoglobulin, lactalbumin, and even caseins [26]. On the other hand, bacterial strains are also known to produce specific peptides to inhibit the growth of other species—for instance, *Lactococcus lactis* is known to produce nisin, *Lactobacillus plantarum* is known to produce plantaricin, etc. In a previous work, we observed that longer fermentation periods were important for achieving the highest lactic acid production, which is also known to be related to higher proteolysis [11]. Here, we used the same industrial starter of LAB species [9,10,11], namely *Lactococcus lactis* subsp. *lactis*, *Lactococcus lactis* subsp. *cremoris*, *Lactococcus lactis* subsp. *lactis* biovar *diacetylactis*, *Streptococcus thermophilus*, and *Lactobacillus delbrueckii* subsp. *bulgaricus*, over 6 days of fermentation, which was one day more than the highest previously observed antibacterial activities [9,10,11]. In short, the milk samples were pasteurized in the factory, and the consortium was added in sterile conditions. After the cheese was prepared, the resulting whey (still containing the LAB consortium) was further fermented for 6 days. The inoculum was well characterized in our previous papers and is an industrial starter. Throughout the 6-day fermentation process, the levels of lactic acid as well as lactose levels were quantified in the obtained whey, as well as the minimal inhibitory activities (determined as the concentration required to obtain a 90% bacterial growth reduction) that the whey samples collected in the 6-day fermentation presented against *L. monocytogenes* and *E. coli* O157. The results are shown in Figure 1 and Table 1, respectively.

**Figure 2:**
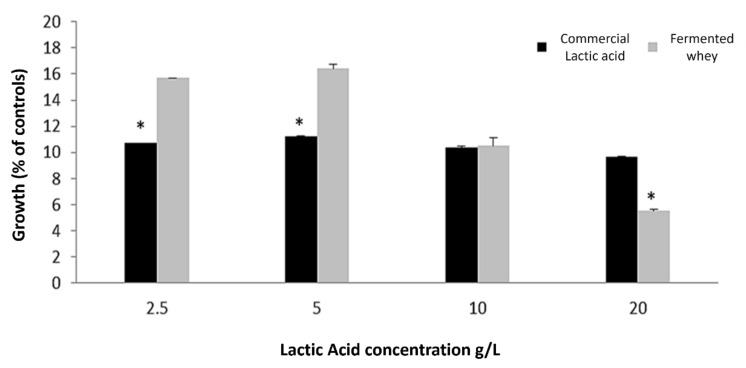
*L. monocytogenes* growth when exposed to different serial dilutions of the 6-day-fermented whey and commercial lactic acid in concentrations equal to those present in the fermented whey at each dilution. Bacterial growth is expressed as % of controls ± SD. * *p* < 0.05.

A steady increase in lactic acid through fermentation was observed, reaching a stable level of 20 g/L after 4 days of fermentation, which is consistent with our previous results [11], but fermentation was observed to continue up to 6 days. However, there was a noticeably significant increase in antibacterial activity on day 6, which was not concomitant to an increase in lactic acid production, suggesting activity related to the presence of other bioactive compounds, namely peptides/polypeptides. In order to confirm whether this activity was not solely due to lactic acid, we compared the antibacterial activity of our 6-day-fermented whey against *L. monocytogenes* in several dilutions against the same amounts of lactic acid alone (Figure 2).

The results in Figure 2 suggest that in the undiluted 6-day-fermented whey (with 20 g/L of lactic acid), the growth inhibition of *L. monocytogenes* was significantly higher (*p* < 0.01) than following exposure to the corresponding concentration of 20 g/L of lactic acid alone. This supports the presence of other antibacterial components in whey besides lactic acid. In the subsequent serial dilutions of whey (corresponding to 10, 5, and 2.5 g/L of lactic acid), this trend was reversed, and lactic acid alone presented the same or even higher inhibitory activities than whey, suggesting that the other antibacterial components in whey become so diluted that they stop exerting an effect.

### 2.1. HPLC Peptide Profile throughout the 6-Day Fermentation

In order to detect and monitor peptide formation, total protein amounts were extracted, and low molecular fractions were obtained through ultrafiltration with cut-offs of 10 and 3 kDa. This allowed the isolation of peptides lower than 10 kDa but higher than 3 kDa, as well as the removal of lactic acid.

**Figure 3:**
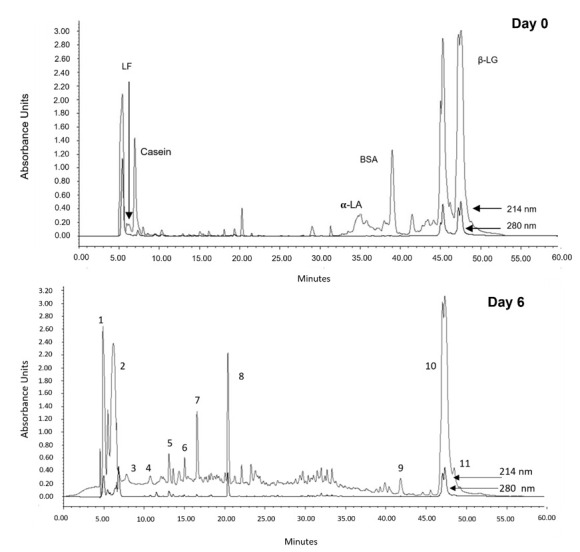
Polypeptide profiles, obtained by RP-HPLC, showing proteolysis and peptide production on day 6 of fermentation with *Lactococcus lactis* subsp. *lactis*, *Lactococcus lactis* subsp. *cremoris*, *Lactococcus lactis* subsp. *lactis* biovar *diacetylactis*, *Streptococcus thermophilus*, and *Lactobacillus delbrueckii* subsp. *bulgaricus.* LF: lactoferrin, α-LA: α-lactalbumin; β-LB: β-lactoglobulin, BSA: bovine serum albumin. Absorbance was read at 214 and 280 nm.

Polypeptides produced through the 6-day fermentation were analyzed using reverse-phase HPLC chromatography. The HPLC profiles of whey fermented in 0 and 6 days are expressed in Figure 3.

For day 0, we can observe the classic peaks corresponding to α-lactalbumin, bovine serum albumin, lactoferrin, and β-lactoglobulin as well as β-casein. Over the fermentation days, these main proteins steadily disappeared (data not shown). Besides BSA and LF proteolysis, the α-LA peak also decreased and was concomitant to the appearance of several peaks with similar hydrophobicity. Most interesting is the fact that the major peaks formed through proteolysis were observed after 6 days of fermentation, which is consistent with our results observed in Table 1 and Figure 1 and Figure 2, suggesting the production of antibacterial polypeptides/peptides at this stage of fermentation. Hence, we further set out to isolate the 11 major peaks from the HPLC separation of day 6, which are marked in Figure 3, and tested their activities against the model bacteria *Escherichia coli* O157 and *Listeria monocytogenes*.

### 2.2. HPLC Peak 2 Presents High Antibacterial Activity

**Figure 4:**
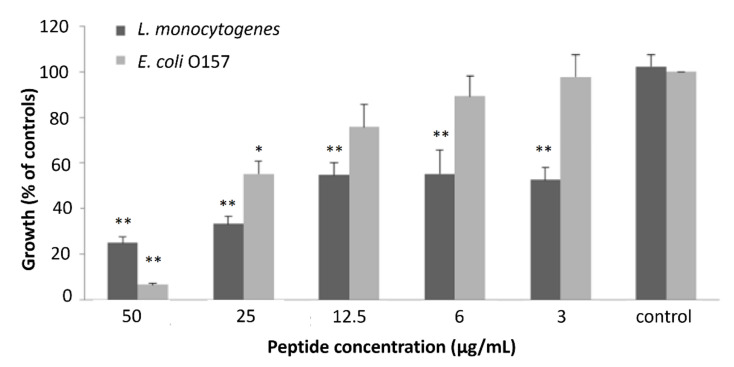
Dose–effect relationship showing inhibition of *L. monocytogenes* and *E. coli* O157 growth induced by different concentrations of HPLC-isolated peak 2. Bacterial growth in each strain is expressed as % of controls ± SD. ** *p* < 0.001, * *p* < 0.05.

Table 2 shows the inhibitory effect of each collected peak of 6-day-fermented whey and the corresponding minimal inhibitory concentrations (MICs) against the growth of the model species *L. monocytogenes* and *E. coli* O157. The results clearly evidence that, although some peaks (peak 1 and peaks 5–11) did not demonstrate antibacterial activity, a few peaks did present strong antibacterial activity, namely peak 2 for both bacteria, peak 3 for *E. coli* O157, and peak 4 for *L. monocytogenes*. Peak 2 clearly stands out as presenting the highest antibacterial activity towards both model bacteria, with a very significantly lower MIC (*p* < 0.001). This antibacterial effect was dose-dependent, as demonstrated by Figure 4. Though effective for both strains, peak 2 seems more effective in reducing the growth of *L. monocytogenes* than that of *E. coli* O157, inducing a higher percentage of growth reduction in all tested concentrations with the exception of 50 µg/mL, where it was more effective against *E. coli* O157.

These results corroborate that the highest antibacterial activities observed on the 6th day of fermentation presented in Table 1 and Figure 2 were attributed not only to lactic acid but also to the formation of bioactive polypeptides. One important aspect to consider is that these fractions were between the molecular masses of 10 and 3 kDa, which would exclude the presence of lactic acid and also of lower molecular mass peptides produced by LAB, such as nisin. Therefore, these polypeptides are most likely the product of the proteolytic activities induced during fermentation. Under this context, peak 2 was selected and further analyzed.

### 2.3. Peak 2 Is A Polypeptide Derived from Beta-Casein Proteolysis

The antimicrobial properties of milk peptides have been widely recognized for many years and are usually reported to derive from lactoferrin; αS1-, αS2-, β-, and κ-casein; α-lactalbumin; β-lactoglobulin, proteosepeptone-3, and lysozyme [8]. Being the polypeptide with the highest activity, and because of the specificity of the LAB consortium, peak 2 was identified by mass spectrometry. The obtained results of peptide homology are presented in Table 3.

According to the MS results, our isolated antibacterial polypeptide digest presents a higher homology to bovine β-casein (Mascot, BLAST, and SWISS-PROT databases).

Although other works have already shown several caseins to be precursors of various peptide fragments with antibacterial activity, most antibacterial casein derivates come from the digestion of α-casein [7]. For example, Zucht et al. (1995) [27] described the isolation and characterization of an antibacterial peptide from bovine milk called casocidin-I. The peptide includes amino acids 165–203 of bovine αS2-casein. Casocidin-I suppressed the growth of Gram-negative (*E. coli*) and Gram-positive (*Staphylococcus carnosus*) bacteria [27]. Nonetheless, fewer studies have shown antimicrobial peptides derived from β-casein. Baranyi et al. (2003) [28] showed antibacterial peptide fragments derived from tryptic digestion of rabbit casein with the sequences of HVEQLLR (residues 50–56 of β-casein), ILPFIQSLFPFAER (residues 64–77 of β-casein), and FHLGHLK (residues 19–25 of α(s1)-casein). These three peptides were synthesized and found to exert antibacterial effects against Gram-positive bacteria only. One other studied β-casein digestion-derived peptide [29] had 26 amino acids and suppressed a large spectrum of Gram-positive and Gram-negative bacteria. One important fact to take into consideration is that although other casein-derived peptides have been determined, this is the first time such a bioactive peptide has been produced by fermentation-induced proteolysis; in the other works, proteolysis was achieved through enzymatic digestion with chymosin or pepsin rather than fermentation [28,29]. It is also important that the MIC found for this polypeptide was extremely low when compared to other β-casein-derived peptides published in other works, showing an MIC of 50 μg/mL [29]. These facts introduce novel possibilities for the use of this antibacterial polypeptide, with the additional advantage that its production involves easy and low-maintenance fermentation that is not only cost-effective but also suggests a high stability at low pH and is possibly resistant to digestion, thus rendering it a good candidate to be easily used in the food industry or as an antibiotic agent. Furthermore, since β-casein and its fragments have been implicated in a number of biological functions, namely in macrophage phagocytosis and peroxide release [30], it is very likely that this polypeptide presents other types of activities as well.

### 2.4. Peak 2 Polypeptide Inhibits Other Pathogenic Bacteria

**Figure 5:**
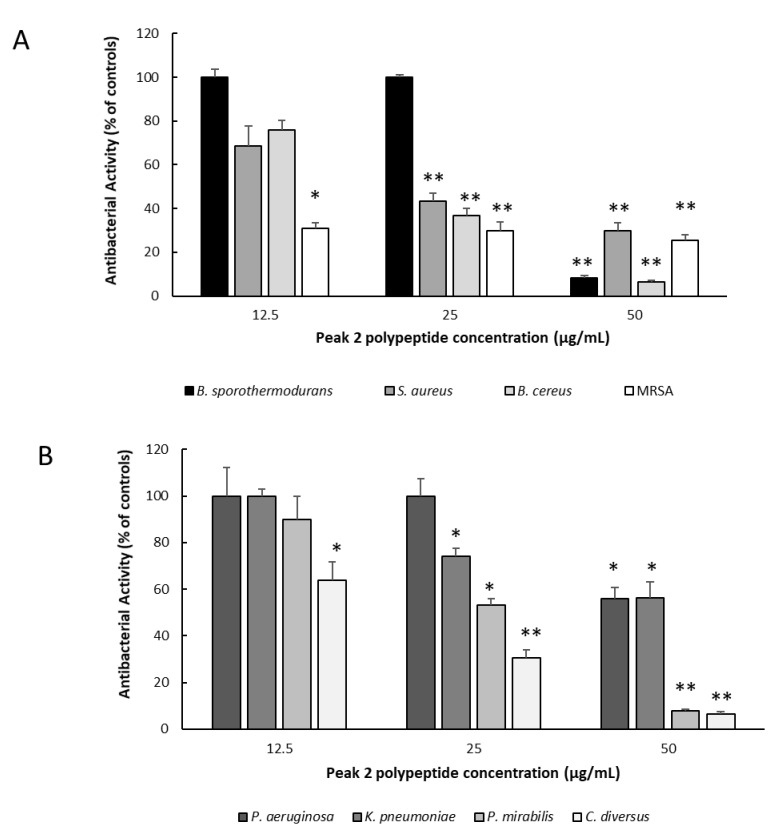
Peak 2 polypeptide antibiotic activity against several pathogens related and not related to food. (**A**) Gram-positive bacteria (*Bacillus cereus*, *Bacillus sporothermodurans*, *Staphylococcus aureus*, and methicillin-resistant *Staphylococcus aureus*); (**B**) Gram-negative bacteria (*Citrobacter diversus*, *Klebsiella pneumoniae*, *Proteus mirabilis*, and *Pseudomonas aeruginosa*). Bacterial growth in each strain is expressed as % of controls ± SD. ** *p* < 0.001, * *p* < 0.05.

Peak 2 was further tested for its antibacterial activity against other types of pathogenic bacteria related and unrelated to food, including methicillin-resistant *Staphylococcus aureus* (MRSA), *Bacillus cereus*, *Bacillus sporothermodurans*, *Staphylococcus aureus*, *Citrobacter diversus*, *Klebsiella pneumoniae*, *Proteus mirabilis*, and *Pseudomonas aeruginosa*. The results are presented in Figure 5.

The isolated peak 2 polypeptide showed various degrees of inhibition against the 8 bacterial strains tested and was able to reduce growth very significantly (*p* < 0.05), in a dose-dependent manner, for almost all bacteria strains. The most effective antimicrobial activity observed with 50 μg/mL of the peptide, in Gram-negative bacteria, were against *P. mirabilis* and *C. diversus* and in Gram-positive bacteria, were against *B. sporothermodurans* and MRSA. These results suggest that the isolated peak 2 has a broad range antibacterial activity, which is not often found in antibacterial peptides from whey. With antibiotic resistance becoming increasingly more a major threat to public health (its predicted that by 2050 antibiotic resistant microorganisms are expected to kill more people than cancer and to cost more money during the next 33 years than the current annual size of the world’s economy [31], these results can be of significant potential. 

### 2.5. Peak 2 Polypeptide Inhibits Gelatinases MMP-2 and -9 and Reduces Colon Cancer Migration

Imbalance of the normal gut microbiota has been strongly linked with gastrointestinal conditions such as inflammatory bowel diseases (IBDs) and irritable bowel syndrome (IBS) [32,33]. Previous results [34] suggested that there may be some activities against MMP-2 and MMP-9 enzymes in 6-day-fermented whey. These two proteases, called gelatinases (MMP-2 and MMP-9), belong to a group of matrix metalloproteinases (MMPs) and are important regulators of the key processes underlying metastasis, including cell adhesion, spreading, migration, invasion, and angiogenesis, and pre-cancer inflammatory diseases such as colitis and inflammatory bowel diseases [16,35]. There is, in fact, a considerably large body of evidence from pre-clinical and clinical tests showing that MMP inhibition reduced cancer growth and metastasization, thus making these gelatinases a good therapeutic target to prevent or reduce colorectal cancer (CRC) [15,16]. Hence, the discovery of a food compound that reduces these gelatinases’ activity is of extreme importance.

Therefore, we further tested the effect of the isolated casein-derived polypeptide on MMP-2 and MMP-9 activity and compared it to the remaining whey, using a standard wound healing assay in colon cancer cells to evaluate possible anti-migration activity and a substrate zymography to determine the anti-gelatinolytic potential of this peptide.

**Figure 6:**
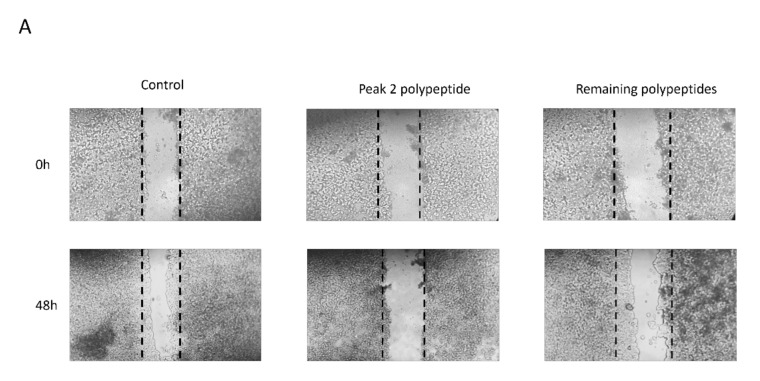

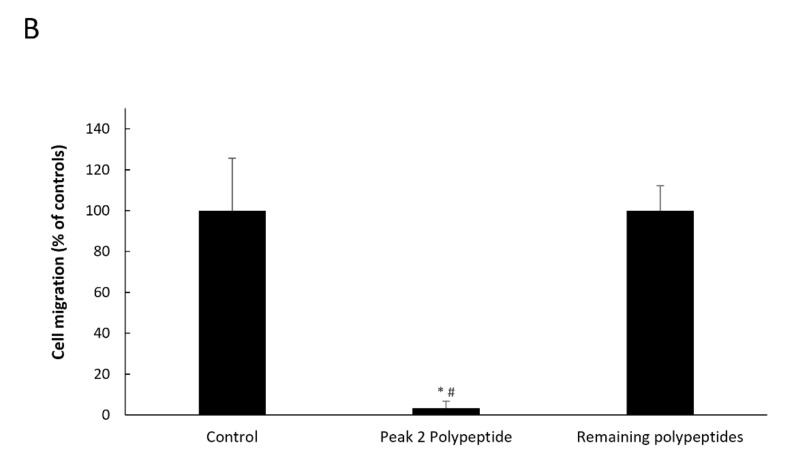
Peak 2 polypeptide inhibits HT29 colon cancer cell migration. (**A**) Examples of cell migration showing the inhibitory effect of different fractions of polypeptides. Cells were grown until reaching 80% confluence, and the monolayer was scratched with a pipette tip (day 0). Cells were then exposed to 100 μg protein/mL of peak 2 polypeptide, and the cell gap invasion was registered after 48 h and compared with the length of the same gap at 0 h. (**B**) Relative migration rates. Values are the means of at least three replicate experiments ± SD and are expressed as % wound closure in relation to day 0. * represents *p* < 0.001 between samples, and # represents *p* < 0.001 when compared to control.

**Figure 7:**
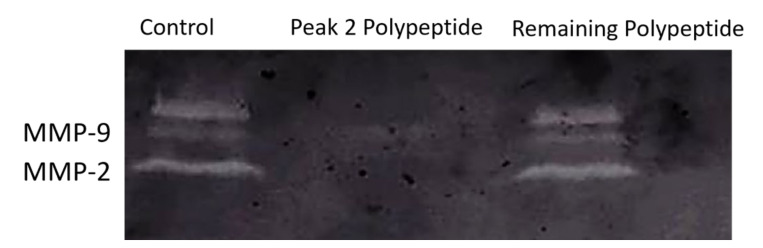
Peak 2 polypeptide inhibits gelatinolytic activity of MMP-2 and MMP-9. Representative image of the zymographic profiles of MMP-9 and MMP-2 activity in the presence of different peptide fractions present in fermented cheese whey. MMP-9 and MMP-2 proteolytic activities were detected in HT29 extracellular media after 48-hour exposure to 100 µg protein/mL.

Figure 6 shows the cell migration patterns of HT29 cells after 48-hour exposure of the studied fractions (peak 2 and the remaining polypeptides from 6-day-fermented whey), and Figure 7 shows the zymographic profiles of the HT29 culture media with the samples tested, where white bands represent gelatinolytic activity and dark bands shows inhibition.

The results obtained clearly demonstrate that the isolated peak 2 polypeptide inhibits MMP-2 and MMP-9 (Figure 7), whilst also significantly (*p* < 0.001) reducing colon cancer cell migration in the HT29 cell line (Figure 6), thus showing the very strong potential of the isolated polypeptide. To our knowledge, this is the first time that such an activity has been reported for fermented cheese whey peptides, although previous studies have described that some peptides from bovine β-casein may inhibit cancer cell growth by stimulating the activity of immune competent cells [36]. In fact, MMP-9 is expressed in a variety of immune cells, e.g., neutrophils, macrophages, lymphocytes, and dendritic cells, as well as in other cells such as osteoblasts, fibroblasts, and endothelial cells [37]. An increase in the expression levels of MMP-9 due to overproduction of these cells has been observed in several types of human cancer as well as in tumor cell lines and animal models [38], which agrees well with our results. With whey polypeptides usually presenting low toxicity and being easy to isolate in larger amounts, this discovery may introduce novel possibilities in the field of cancer treatment or prevention. Additionally, the peak 2 polypeptide is produced from cheese whey fermentation with LAB, which further holds the advantage of being a probiotic with high nutritional value since it contains all the essential amino acids of milk without the risk of food intolerance or allergies. Particularly now, with milk being suggested to induce cancer and inflammation [38,39], the discovery of a dairy-derived polypeptide from a byproduct of cheese manufacturing with anti-cancer, anti-inflammatory, and antibacterial effects can have strong industrial, social, health, and economic potential.

Overall, this is, to our knowledge, the first time a peptide, particularly arising from beta casein, with strong antibacterial activities is also an MMP inhibitor. The combination of antibacterial activity and anti-inflammatory potential can have great use for diets related to chronic diseases such as inflammatory bowel diseases, which are also related to MMP-9 activity, gut pathogens, and the microbiome. With the increasing health concerns about multi-drug-resistant bacterial diseases, gut inflammatory diseases, gut microbiome, and foodborne pathogens, this peptide could indicate a turning point in the field of health foods and nutrition and could be used in preventive/curative diets.

## 3. Materials and Methods

### 3.1. LAB and Media

An industrial bacterial starter mix (Danisco, Sassenage, France) of *Lactococcus lactis* subsp. *lactis*, *Lactococcus lactis* subsp. *cremoris*, *Lactococcus lactis* subsp. *lactis* biovar diacetylactis, *Streptococcus thermophilus*, and *Lactobacillus delbrueckii* subsp. *bulgaricus* containing approximately 7 log_10_ CFU/mL of whey after cheese manufacturing was kept at −18 °C to be used as necessary for fermentation assays. Bacterial cell evaluation was performed on Man Rogosa and Sharp medium modified (MRS-L) by the addition of 2% (m/v) lactose (BD Difco, Becton, Dickinson and Company, Franklin Lakes, NJ, USA) and containing 1% (m/v) peptone (Biokar Diagnostics, Beauvais, France), 1% (m/v) meat extract (Biokar Diagnostics, Beauvais, France), 1% (m/v) yeast extract (Biokar Diagnostics, Beauvais, France), 1 mL Tween 80, 2% (m/v) dipotassium hydrogen phosphate (Merck, Darmstadt, Germany), 5% (m/v) sodium acetate (BDH, VWR, Lisbon, Portugal), 2% (m/v) diammonium citrate (Sigma-Aldrich, Merck, Darmstadt, Germany), 0.2% (m/v) magnesium sulphate, 0.05% (m/v) manganese, and 2% (m/v) agar (Oxoid, Basingstoke, UK), prepared according to Man et al. (1960) [40]. Incubation was performed in anaerobic jars (AnaeroPack, Mitsubishi, Tokio, Japan) with a gas generation device (Genbox Anaer, bioMérieux, Marcy l’Étoile, France) at 37 °C for 48 h.

### 3.2. Whey Fermentation Conditions

Whey obtained after the manufacturing of cheese from a mixture of ewe, goat, and cow milk, containing 0.04% (m/v) NaCl, pH 6.65 (Lab 850, Schott AG, Mainz, Germany), and 30 g/L of lactose, quantified through ionic exchange in high-performance liquid chromatography (HPLC) as previously described [9], was used for fermentation assays.

Whey in natura was divided into aliquots of 500 mL and distributed into Erlenmeyer flasks and placed into an incubator at 37 °C for 6 days.

### 3.3. Evaluation of Fermentation

Measurements of pH (Lab 850, Schott AG, Mainz, Germany), lactic acid production, and consequent lactose consumption were quantified throughout 6 days of fermentation, the latter two by ionic exchange in an HPLC System (Waters Corporation, Milford, MA, USA) equipped with a 717 plus Autosampler (Waters Corporation, Milford, MA, USA), a 515 HPLC Pump (Waters Corporation, Milford, MA, USA), and a Refractive Index Detector (RID) (486 Waters Corporation, Milford, MA, USA) for compound detection, as previously described [23]. Prior to injection, samples were centrifuged at 10,000 g (Eppendorf 5414D, Hamburg, Germany) for 10 min, and the supernatants were filtered through a Millipore membrane (Merck, Darmstadt, Germany) with a pore size of 0.2 µm. Samples were injected in a Schodex SUGAR SH1011 column (Waters Corporation, Milford, MA, USA), and separations were achieved at 50 °C, using 5 mM sulfuric acid as the mobile phase (isocratic elution) at a flow rate of 0.6 mL/min. Calibration curves were made with standard solutions (in 5 mM sulfuric acid) of lactose (Sigma-Aldrich, Netherlands) and lactic acid (Sigma-Aldrich, Netherlands). Peak integration was performed using the HPLC software Empower Pro (Waters Corporation, Milford, MA, USA).

### 3.4. Protein Quantification

The protein content in each sample was determined using the modified Lowry method [41], with bovine serum albumin as the standard. In the case of the isolated peptide fractions, samples were freeze-dried (Modulyo, Edwards, UK), and their respective dry weights were measured.

### 3.5. Peptide Isolation through Ultrafiltration

Collected whey fractions were subjected to denaturation by boiling at 100 °C in order to precipitate non-hydrolyzed proteins. Samples were subsequently centrifuged at 10,000× *g*, and the supernatants were fractionated by ultrafiltration using a “VivaSpin” (Vivaproducts, Littleton, MA, USA) with a molecular weight cut-off (MWCO) of 10 kDa and centrifuged at 1400× *g*, 4 °C. Peptide fractions were subsequently desalted by ultrafiltration using a “VivaSpin” (Vivaproducts, Littleton, MA, USA) with an MWCO of 3 kDa and centrifuged at 1400× *g*, 4 °C.

### 3.6. Peptide Separation through HPLC

Whey samples were separated in an HPLC device (Waters 2695 Separations Module, Waters Corporation, Milford, MA, USA) equipped with a Waters 2998 Photodiode Array Detector (Waters Corporation, Milford, MA, USA). Peptides were separated in a C18 column (Zorbax 300SB 5 µm, 250 × 4.6 mm, Agilent Technologies, Santa Clara, CA, USA). Elution was conducted with eluant A (0.1% (*v*/*v*) TFA, Sigma-Aldrich, Merck, Darmstadt, Germany) and solvent B (Acetonitrile in 0.1%, Sigma-Aldrich, Merck, Darmstadt, Germany, (*v*/*v*) TFA). Peak detection was performed at 214 and 280 nm. Whey protein detection was performed with commercial standards.

### 3.7. Antibacterial Activity of the Peptide Fractions

Peptide fractions were tested for their antibacterial activity against the model bacteria *E. coli* O157 and *L. monocytogenes*. Minimal inhibitory concentrations (MICs) were assessed in sterile 96-well plates (Greiner Bio-one, Frickenhausen, Germany) using the microdilution method as described by Bouhdid et al. (2010) [42]. Briefly, 50 μL of Müller–Hinton medium (Biokar Diagnostics, Beauvais, France) was added to each well, and 50 μL of sample was added to the first well and serially diluted at a 1:2 ratio in each adjacent well for 10 dilutions. Subsequently, 50 μL of the bacterial suspension with a concentration of 2 × 10^5^ CFU/mL was added to the wells. A positive control (50 μL of Müller–Hinton medium + 50 μL bacterial suspension) and a negative control (100 μL Müller–Hinton medium) were performed. The plates were incubated for 24 h at 37 °C, and the absorbance was read at 546 nm (Synergy HT, Biotek, Winooski, VT, USA) at the beginning of the inoculation and at the end of the assay.

The peak 2 peptide was further tested for its antibacterial activity against Gram-positive (*Bacillus cereus*, *Bacillus sporothermodurans*, *Staphylococcus aureus*, and methicillin-resistant *Staphylococcus aureus*) and Gram-negative (*Citrobacter diversus*, *Klebsiella pneumoniae*, *Proteus mirabilis*, and *Pseudomonas aeruginosa*) bacteria. These activities were performed as described above.

### 3.8. Mass Spectrometry (MS) Analysis

Selected isolated peaks were analyzed on a 5600 TripleTOF (ABSciex^®^, Framingham, MA, USA) in information-dependent acquisition (IDA) mode. Peptides were resolved by liquid chromatography (nanoLC Ultra 2D, Eksigent^®^, McKinley Scientific, Cary NC) on a MicroLC column ChromXPTM C18CL reverse phase column (300 μm ID × 15 cm length, 3 μm particles, 120 Å pore size, Eksigent^®^, McKinley Scientific, Cary NC) at 5 μL/min. Peptides were eluted into the mass spectrometer with a multistep gradient: 0–2-minute linear gradient from 5 to 10%, 2–45-minute linear gradient from 10 to 30%, and 45–46 min to 35% acetonitrile in 0.1% FA. Peptides were eluted into the mass spectrometer using an electrospray ionization source (DuoSpray™ Source, AB Sciex, Framingham, MA, USA) with a 50-micrometer internal diameter (ID) stainless steel emitter (New Objective).

For information-dependent acquisition (IDA) experiments, the mass spectrometer was set to scan full spectra (350–1250 m/z) for 250 ms, followed by up to 100 MS/MS scans (100–1500 m/z from a dynamic accumulation time—minimum 30 ms for precursor above the intensity threshold of 1000—in order to maintain a cycle time of 3.3 s). Candidate ions with a charge state between +2 and +5 and counts above a minimum threshold of 10 counts per second were isolated for fragmentation, and one MS/MS spectrum was collected before adding those ions to the exclusion list for 25 s (mass spectrometer operated by Analyst^®^ TF 1.6, ABSciex^®^, Framingham, MA, USA). Rolling collision was used with a collision energy spread of 5. Two IDA experiments were performed for each sample, with the second analysis performed with an exclusion list of the peptides previously identified.

### 3.9. Polypeptide Identification

Protein identification was performed using Protein Pilot™ software (v 5.0, ABSciex^®^, Framingham, MA, USA) with the following search parameters: identification from the UniProt database from March 2016, with no alkylation or digestion for the peptide samples P1, P2, and P3. As criteria for protein filtering, we used an unused score value of 1.3, 95% peptide confidence filtering, and >0 contribution.

### 3.10. In Vitro Colon Cancer Cell Assays

#### 3.10.1. HT29 Cell Cultures

The human colon adenocarcinoma cell line HT29 (ECACC 85061109), obtained from a 44-year-old Caucasian female, was used. HT29 cells were maintained according to Lima, Mota, Monteiro, and Ferreira (2016) [43].

#### 3.10.2. Wound Healing Assay

For cell migration analysis, a wound healing assay was performed according to the procedure described previously [43]. HT29 cells (5×10^5^ cells/well) were seeded in 24-well plates and allowed to reach 80% confluence. Each well was subsequently supplemented with fresh medium containing two protein fractions (peak 2 peptide and other peptides, both present on day 6 of fermentation) at a concentration of 100 μg/mL. The invaded area after 48 h was calculated in each treatment and compared to the initial area at 0 h. Results are presented as the mean ± SD (when compared to the control).

#### 3.10.3. MMP-9 and MMP-2 Catalytic Activity

To determine the specific metalloproteinase activities expressed in the culture media, gelatin zymography was performed according to Toth, Sohail, and Fridman (2012) [44] and Lima, Mota, Monteiro, and Ferreira (2016) [43].

### 3.11. Statistical Analysis

Analysis of variance (one-way ANOVA) was used to assess significant differences between samples at a significance level of 95% (*p* < 0.05). Multiple comparisons were performed through Tukey’s test using SigmaStat software.

## 4. Conclusions

The results presented here demonstrate that the LAB consortium and the fermentation used on whey from a mixture of milks (ewe, cow, and goat) produce a specific novel β-casein-derived bioactive polypeptide with an unusual and potent dual antibacterial and anti-gelatinase bioactivity. Overall, this is, to our knowledge, the first time a peptide from whey has been reported to have this combined activity, which opens doors to novel dietary approaches to prevent/treat chronic diseases such as inflammatory bowel diseases, which are also related to MMP-9 activity and gut pathogens. Since it also exhibits high stability under high-salt, low-pH, and high-temperature conditions, it is most likely resistant to digestion, making this polypeptide a good candidate to be used in the formulation of antibacterial nutraceuticals; for food preparations, with its disinfecting and antibiotic properties; or for IBD prevention and promotion of a healthier lifestyle. With the current problematics involving multi-drug-resistant bacterial diseases, gut inflammatory diseases, the gut microbiome, and foodborne pathogens, our results could indicate a turning point in the field of health foods and nutrition, and this peptide could be used in preventive/curative diets.

## Figures and Tables

**Table 1:** Minimal inhibitory concentrations (MICs) of whey samples, determined against the model species *L. monocytogenes* and *E. coli* O157. Whey was collected throughout the 6 days of whey fermentation and was tested using the microdilution method. MICs were determined as the concentration required to reduce bacterial growth to 90%. Bacterial growth was determined by absorbance at 546 nm, and percentages were determined as a percentage of controls (bacterial growth with no whey present). Values are expressed in µg/mL for each whey sample collected. ND = not detected.

	MIC (μg/mL)	
Days of Fermentation	*L. monocytogenes*	*E. coli* 0157
**1**	ND	25.0
**2**	ND	25.0
**3**	ND	25.0
**4**	25.0	25.0
**5**	25.0	25.0
**6**	0.20	0.40

**Table 2:** Minimal inhibitory concentrations (MICs) of the different peaks collected by HPLC separation of the day 6 fermented whey against the growth of the model species *L. monocytogenes* and *E. coli* O157. MICs were determined as the concentration required to induce 90% bacterial growth. Values are expressed in µg/mL for each peak collected. ND = not detected for the highest concentration used in this assay (50 µg/mL).

MIC (μg/mL)											
	Peak 1	Peak 2	Peak 3	Peak 4	Peak 5	Peak 6	Peak 7	Peak 8	Peak 9	Peak 10	Peak 11
***L. monocytogenes***	ND	3	ND	50	ND	ND	ND	ND	ND	ND	ND
***E. coli***	ND	6	50	ND	ND	ND	ND	ND	ND	ND	ND

**Table 3:** Results of the MS analysis polypeptide from peak 2. Matched peptides were identified with more than 95% confidence.

N	Name	Peptides (95%)
**1**	Beta-casein OS=Bos taurus GN=CSN2 PE=1 SV=2	30
